# Effects of Nutrients in Substrates of Different Grains on Aflatoxin B_1_ Production by* Aspergillus flavus*

**DOI:** 10.1155/2016/7232858

**Published:** 2016-05-17

**Authors:** Jie Liu, Lvhui Sun, Niya Zhang, Jiacai Zhang, Jiao Guo, Chong Li, Shahid Ali Rajput, Desheng Qi

**Affiliations:** Department of Animal Nutrition and Feed Science, College of Animal Science and Technology, Huazhong Agricultural University, Wuhan, Hubei 430070, China

## Abstract

The current study was to better understand the potential factors affecting aflatoxin B_1_ (AFB_1_) accumulation varies between different grains. The nutrient composition and contents of defatted substrates were determined; additionally, according to the nutrient content of the substrates, the effects of starch, soluble sugars, amino acids, and trace elements on AFB_1_ production and mycelial growth in Czapek-Dox medium were examined. These results verified that removal of lipids from ground substrates significantly reduced the substrate's potential for AFB_1_ production by* Aspergillus flavus*. Maltose, glucose, sucrose, arginine, glutamic acid, aspartic acid, and zinc significantly induced AFB_1_ production up to 1.7- to 26.6-fold. And stachyose more significantly promoted* A. flavus* growth than the other nutrients. Thus, this study demonstrated that, combined with the nutrients content of grains, in addition to lipids, sucrose, stachyose, glutamic acid, and zinc might play key roles in various grains that are differentially infected by* A. flavus*. Particularly, two new nutrients (arginine and stachyose) of the grains we found significantly stimulate AFB_1_ production and* A. flavus* growth, respectively. The results provide new concepts for antifungal methods to protect food and animal feed from AFB_1_ contamination.

## 1. Introduction

Maize, wheat, soybean, and peanut are the major economic crops in most countries. These grains and their by-products (e.g., soybean meal, peanut meal, and corn germ meal) are used extensively for food and animal feed [1]. In addition, these products provide the best natural substrate for mould, which can be easily contaminated with mycotoxins under suitable conditions [2, 3]. Recently, the problem of food and animal feed contamination with mycotoxin, particularly aflatoxin contamination, has received attention worldwide [4, 5].

Aflatoxins are a secondary metabolite produced by* A. flavus* and* A. parasiticus*. These toxins mainly exist in four forms in nature: aflatoxin B_1_ (AFB_1_), aflatoxin B_2_, aflatoxin G_1_, and aflatoxin G_2_. AFB_1_ was classified as a group I human carcinogen by IARC [6]. Thus, the AFB_1_ contamination of crops and by-products poses a serious threat to human and animal health and is associated with enormous economic losses in agricultural economy and animal husbandry [7].

Most previous studies focus on controls on AFB_1_ production in crops; however, few studies concerned the factors for the different AFB_1_ contamination in various grains. Although several researches reported that there was a relationship between lipid and AFB_1_ production in crops [8–12], because of the complex compositions of grains, AFB_1_ production not only is related to the lipids but also is associated with other nutrients, such as starch, proteins, saccharides, and trace elements. Our previous study suggested that, in addition to the lipid, the nutrient composition and content of the substrates might be the key factors affecting AFB_1_ production by* A. parasiticus* [13]. Recently, some studies reported that some nutrients were related to AFB_1_ biosynthesis. For example, a strong relationship between maize endosperm starch and AFB_1_ contamination was previously hypothesized [14, 15]. A genetically modified maize crop capable of inhibiting *α*-amylase activity was recommended for AFB_1_ contamination control [16]. However, the maize whole-kernel study of Mellon et al. [12] did not support this hypothesis. On the other hand, glucose, ribose, xylose, and glycerol were considered excellent substrates for both growth and aflatoxin production by* A. parasiticus* [17]. Mellon et al. [12] also reported that* A. flavus* utilizes saccharides as the basic carbon source for mycelial growth and AFB_1_ production. In addition, Payne and Hagler Jr. [18] found that proline stimulated aflatoxin production more than asparagine, but Reddy et al. [19] reported that asparagine excellently supported the production of aflatoxin. Meanwhile, trace elements can also affect AFB_1_ biosynthesis. Lillehoj et al. [20] reported that trace element contents were higher in AFB_1_-contaminated maize germ compared with noncontaminated maize germ. Furthermore, Stossel [21] found that zinc supplementation could promote AFB_1_ biosynthesis in soybean, and Cuero et al. [22] reported that iron, copper, and zinc induced aflatoxin production by* A. flavus*. These previous studies just focused on the effects of nutrients on the production of aflatoxin by* A. flavus* and* A. parasiticus*. However, few studies researched on the relationships between the various nutrients in grains and the differential AFB_1_ contamination of these grains.

The objectives of this study were to examine differences in the nutrient composition and content of four grains (maize, wheat, soybean, and peanut) and corn isolate tissues and according to the nutrients content of grains and isolate tissues to systematically evaluate the effects of different nutrients (lipids, amino acids, starch, soluble sugars, and trace elements) on* A. flavus* growth and AFB_1_ biosynthesis by* A. flavus*, to identify the nutrients responsible for the different AFB_1_ contamination in these grains and isolate tissues. Taken together, these results provide new concepts for antifungal methods to protect food from AFB_1_ contamination.

## 2. Materials and Methods

### 2.1. Samples and Reagents

Four samples (corn, wheat, peanut, and soybean) were collected from raw material manufacturers in China, and two samples (corn germ and corn endosperm) were obtained by manual dissection [12]. All samples were ground to a fine, 40-mesh powder using a laboratory mill. The samples were stored at −20°C in zipper bags prior to use. A total of six soluble sugars (stachyose, raffinose, sucrose, fructose, maltose, and glucose), five amino acids (aspartic acid, glutamic acid, arginine, alanine, and glycine), and corn oil were purchased from Aladdin Industrial Corporation (Shanghai, China) and stored at 4°C.

### 2.2. Fungal Cultures and Preparation of Spore Suspensions

*A. flavus* isolate (NRRL-3357) was used in this study with a known AFB_1_ production capacity [23] and it is widely used in laboratory and field studies in B group (B_1_ and B_2_) aflatoxins and mainly product aflatoxin B_1_. The isolate was maintained as a glycerol stock preparation at −80°C. It was grown on potato dextrose agar (E. Merck) medium at 30°C for 7 days. Mature spores were harvested with sterile 0.05% Tween 80 saline solution [24]. Spore suspensions were diluted to approximately 2 × 10^7^ spores/mL or 1 × 10^8^ spores/mL. The spore population was quantified using a haemocytometer.

### 2.3. Effects of Corn Oil and Different Substrates on AFB_1_ Production in* A. flavus*

The initial crude fat of full-fat substrates was determined using the solvent extraction method [25], and ground peanut, soybean, corn, wheat, corn germ, and corn endosperm were defatted in a Soxhlet apparatus with anhydrous diethyl and stored at −20°C in zipper bags until further use.

Twenty grams of each substrate (full-fat peanut, soybean, corn, wheat, corn germ, and corn endosperm; defatted peanut, soybean, corn, wheat, corn germ, and corn endosperm) was added to a series of 150 mL Erlenmeyer flasks, which were autoclaved at 121°C for 20 min and then cooled. Corn oil was added to the corresponding flask at a rate of 10 g/100 g of defatted substrates and full-fat substrates without corn oil. All treatments consisted of five replicates, and each experiment was repeated three times. Inoculated treatments received 2 mL of an* A. flavus* conidial suspension (2 × 10^7^ spores/mL) and additional sterile deionized water to adjust the moisture to 25%. Initial moisture was determined using the oven-drying method [26]. All treatments were incubated at 30°C and 85% relative humidity. Samples were collected on the 15th day to determine AFB_1_ production.

### 2.4. Determination of the Starch, Soluble Sugar, Amino Acid, and Trace Element Contents of Samples

Starch content was determined using the enzyme hydrolysis method [27]. Soluble sugar extraction was performed according to Mellon et al. [12] with slight modifications. Briefly, soluble sugar analysis was performed by adding 0.2 g of ground corn sample to 2.0 mL of deionized water, allowing the mixture to sit for 5 min, and then vortex mixing for 1 min. This soak/mix cycle was repeated twice, and the sample was centrifuged at 2000 g for 5 min. The supernatant was removed and stored at −20°C. Prior to drying, the sample was centrifuged at 1500 g for 5 min, and the supernatant was filtered through a 0.22 *μ*m Millex PTFE (Tianjin, China). Next, 0.5 mL of this filtrate was evaporated under nitrogen at 50°C. The soluble sugar analysis method was performed according to Mellon et al. [28]. The amino acid hydrolysis was performed according to GB/T 18246-2000 [29], and then, the solution was filtered through a 0.22 *μ*m PTFE filter for amino acid analysis by liquid chromatography, as previously described by Gratzfeld-Huesgen [30]. The trace element content was detected using atomic absorption spectrometry [31].

### 2.5. Effects of Starch, Soluble Sugars, Amino Acids, and Trace Elements on AFB_1_ Production by* A. flavus*

The basic medium used in this study was Czapek-Dox medium (0.3% NaNO_3_, 0.1% K_2_HPO_4_, 0.05% MgSO_4_·7H_2_O, 0.05% KCl, 0.001% FeSO_4_·7H_2_O, and 3.0% sucrose). Next, 30 mL of basic medium was added to a series of 100 mL Erlenmeyer flasks. Five amino acids (aspartic acid, glutamic acid, arginine, alanine, and glycine) were added to the corresponding flask at rates of 0.5, 1.0, and 2.0 g/100 mL. Four trace elements—copper (Cu, CuSO_4_·5H_2_O), iron (Fe, FeSO_4_·7H_2_O), zinc (Zn, ZnSO_4_·7H_2_O), and manganese (Mn, MnSO_4_·H_2_O)—were added to the corresponding flask; the concentrations of Cu, Fe, Zn, and Mn were 5, 10, 20, and 50 mg/L; 20, 50, 100, and 200 mg/L; 20, 50, and 100 mg/L; and 10, 20, 50, and 100 mg/L, respectively. The concentrations of starch were 0.5, 1.0, 2.0, 3.0, and 5.0 mg/L, and starch was substituted for sucrose in the basic medium. The basic medium served as a control for the three experiments. For soluble sugar incubation, six soluble sugars (stachyose, raffinose, sucrose, fructose, maltose, and glucose) were substituted for sucrose in the basic medium, and the concentrations of soluble sugars were 0.5, 1.0, 3.0, and 6.0 g/100 mL. The media were adjusted to pH 6.0 using 6 M HCl or 5 M NaOH. Inoculated treatments received 100 *μ*L of an* A. flavus* conidial suspension (1 × 10^8^ spores/mL). All treatments were maintained at 30°C with shaking at 150 rpm for 5 days. AFB_1_ was extracted from the liquid medium. The experiment used a total of five replicates and was performed three times. The mycelia were collected by filtration and washed with distilled water three times and then dried for 48 h at 60°C to determine the total mycelia biomass [32].

### 2.6. AFB_1_ Extraction and Analysis

Substrate samples were extracted to determine the AFB_1_ production according to the method of Ma et al. [13]. Next, 15 mL fluid medium samples were extracted three times with 20 mL (10, 5, and 5 mL) of chloroform, and the extract was evaporated under nitrogen at 60°C. The residue was stored at 4°C for AFB_1_ detection.

The residue was redissolved in 200 *μ*L of acetonitrile/water (9 : 1, v/v) and then derivatized using 700 *μ*L of TFA (trifluoroacetic acid)/acetic acid/water (20 : 10 : 70, v/v/v), as described by Trucksess et al. [33]. Fifty microliters of derivatized solution was filtered through Millex PTFE 0.22 *μ*m filters (Tianjin, China) and analysed using a reversed-phase HPLC/fluorescence detection system by Abdel-Hadi et al. [34] with slight modifications. Sample extracts were analysed using an Agilent 1260 series HPLC (Waldbronn, Germany) equipped with a fluorescence detector (*λ*_exc_ 360 nm; *λ*_em_ 440 nm) and a C18 column (250 × 4.6 mm, 5 *μ*m; Agilent). The analysis was performed using a mobile phase of water: methanol: acetonitrile (60 : 30 : 10) at a flow rate of 1 mL/min and a run time of 20 min. The temperature of the column oven was 30°C. The mean recovery percentage for AFB_1_ exceeded 90%. The limit of detection of the analytical method was 1 ng·g^−1^.

### 2.7. Statistical Analysis

All data analyses were performed by analysis of variance (ANOVA) using the SPSS statistical analysis system software (SPSS 22, Chicago, USA) and presented as the mean ± standard deviation (SD). Significant differences were identified using Duncan's new multiple range test at the *P* = 0.05 or *P* = 0.01 level.

## 3. Results

### 3.1. Effects of Oil and Different Substrates on AFB_1_ Production in* A. flavus*

Table 1 showed that the six defatted substrates were slightly contaminated by* A. flavus* and had relatively low levels of AFB_1_ accumulation. However, AFB_1_ accumulation sharply increased with the addition of corn oil. The concentrations of AFB_1_ in full-fat substrates were also higher than in the defatted substrates. Specifically, when corn oil was added to defatted wheat, the AFB_1_ concentration (14.87 mg/kg) increased 495- (0.03 mg/kg) and 135-fold (0.11 mg/kg) compared to defatted wheat and full-fat wheat, respectively. Other groups also showed the same trend, except the corn germ and soybean group which have abundant lipids in full-fat substrates. However, different substrates have different effects on AFB_1_ production. Avoiding the effect of lipids, when substrates were defatted, the maximum AFB_1_ concentrations were found in defatted soybean (8.15 mg/kg), followed by defatted corn germ, peanut, corn endosperm, corn, and wheat.

### 3.2. Starch, Soluble Sugars, Amino Acids, and Trace Elements Contents of Different Substrates

The four main nutrients (starch, soluble sugars, amino acids, and trace elements) of defatted substrates were examined. The nutrients composition and content of six defatted substrates were very different. The starch contents of defatted wheat, corn, and corn endosperm were 67.22%, 72.21%, and 82.19%, respectively, and were significantly higher than those of the other three substrates (Table 2).

The concentrations of six soluble sugars (fructose, glucose, sucrose, maltose, raffinose, and stachyose) are shown in Table 2. Soluble sugars were most abundant in defatted corn germ, followed by defatted soybean and peanut. Specifically, there were high concentrations of sucrose in defatted corn germ (42.36 mg/g), soybean (31.17 mg/g), and peanut (32.50 mg/g). Furthermore, the stachyose content was particularly high in defatted soybean (34.71 mg/g) (*P* < 0.01).

This study determined the contents of sixteen amino acids, and based on previous studies and differences in the amino acid contents, only five amino acids contents are shown in Table 2; the others are not shown. The protein content was the sum of the contents of the sixteen amino acids analysed. Among the studied amino acids, glutamic acid had the highest content, followed by aspartic acid. Although the other three amino acid contents were different, these differences were not obvious compared with the contents of glutamic acid and aspartic acid. The amino acid contents of defatted peanuts and soybeans were relatively high compared with those of other grains and isolate tissues. Specifically, the glutamic acid contents of defatted soybean and peanut were 10.56% and 13.89%, respectively.

The trace element contents of different grains were fairly different. The maximum content of Cu was 34.63 mg/kg in defatted peanut, and its minimum content (6.89 mg/kg) was observed in defatted corn. The maximum contents of Fe and Zn were found in defatted corn germ (145.05 and 107.47 mg/kg), and their minimum contents were identified in defatted corn endosperm. The defatted peanut and wheat were rich in Mn. Thus, the content of the four trace elements was the most abundant in defatted corn germ, followed by defatted soybean and peanut, whereas it was minimal in corn endosperm (Table 2).

### 3.3. Effects of Starch, Soluble Sugars, Amino Acids, and Trace Elements on AFB_1_ Production

According to the solubility of the starch, this study selected five concentrations from 0.5% to 5.0%, and basal medium served as a control. These results showed that starch did not promote AFB_1_ production and inhibited AFB_1_ synthesis. The AFB_1_ content of control was 194.4 ng/30 mL, but in the five treatments, the maximum content of AFB_1_ was only 7.64 ng/30 mL which was found in 2.0% starch.

The effect of soluble sugars on AFB_1_ production is shown in Table 3. The soluble sugar addition concentrations depend on the contents of the six substrates. These results indicated that low concentrations of soluble sugars could not promote AFB_1_ biosynthesis; however, when the soluble sugar concentration reached 3.0% and 6.0%, the soluble sugars promoted AFB_1_ production somewhat, including glucose, sucrose, and maltose (*P* < 0.05). When the concentration was 3.0%, sucrose exhibited the highest promoting effect on AFB_1_ production (39782.61 ng/30 mL), followed by maltose (23687.29 ng/30 mL) and fructose, which had the smallest effect. However, when the concentration was 6.0%, the enhancing effects of sucrose (4471.97 ng/30 mL), raffinose (5.50 ng/30 mL), and stachyose (120.61 ng/30 mL) were reduced. Maltose promoted aflatoxin production to the greatest extent (74848.68 ng/30 mL), followed by glucose (35860.57 ng/30 mL). Among the six soluble sugars, raffinose exhibited the weakest enhancement.

**Figure 1:**
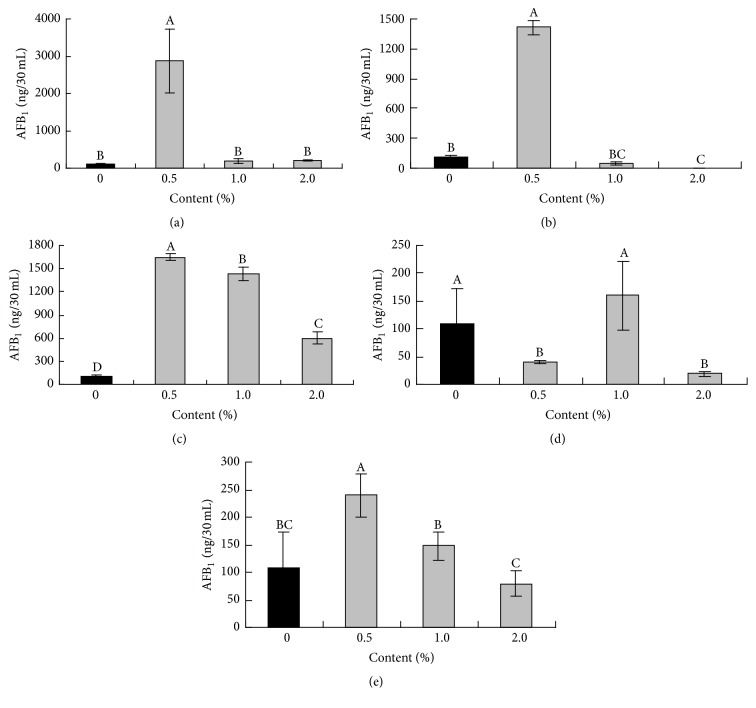
Effects of glutamic acid (a), aspartic acid (b), arginine (c), alanine (d), and glycine (e) on AFB_1_ accumulation (ng/30 mL) at concentrations of 0, 0.5, 1.0, and 2.0 g/100 mL with incubation on a constant temperature shaker (30°C) for 5 days. The data represent the mean ± SD. Different superscript letters represent significant differences at *P* < 0.05, and all treatments consisted of five replicates.

The amounts of amino acid added in this study were based on the concentrations used by Payne and Hagler Jr. [18] and amino acid concentrations of the substrates (Table 2). As the amount of amino acids increased, aflatoxin synthesis decreased (Figure 1). However, only 0.5% glutamic acid, aspartic acid, and glycine significantly stimulated AFB_1_ production (*P* < 0.05), whereas all concentrations of arginine significantly stimulated AFB_1_ production (*P* < 0.05). Moreover, 0.5 and 2.0% alanine reduced AFB_1_ production.

Different trace elements were selected to obtain different gradient concentrations depending on the trace element contents of the six substrates (Table 4). Only zinc significantly promoted AFB_1_ biosynthesis, and as the zinc concentration increased, AFB_1_ accumulation also increased. When the concentrations of zinc were 20, 50, and 100 mg/L, the contents of AFB_1_ were increased 4-, 5-, and 19-fold, respectively. In addition, the other three trace elements significantly inhibited AFB_1_ production (*P* < 0.05) at all concentrations examined.

### 3.4. Effects of Starch, Soluble Sugars, Amino Acids, and Trace Elements on* A. flavus* Growth

**Figure 2:**
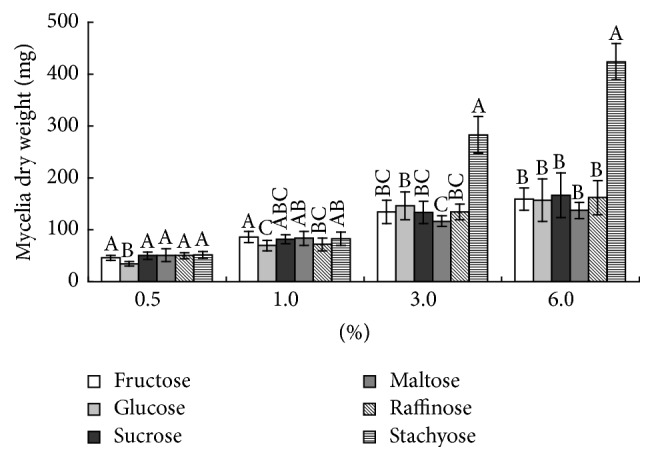
Effects of soluble sugars on* A. flavus* growth. Different superscript letters represent significant differences at *P* < 0.05 for each soluble sugar. Error bars show standard deviations of the results of five replications.

**Figure 3:**
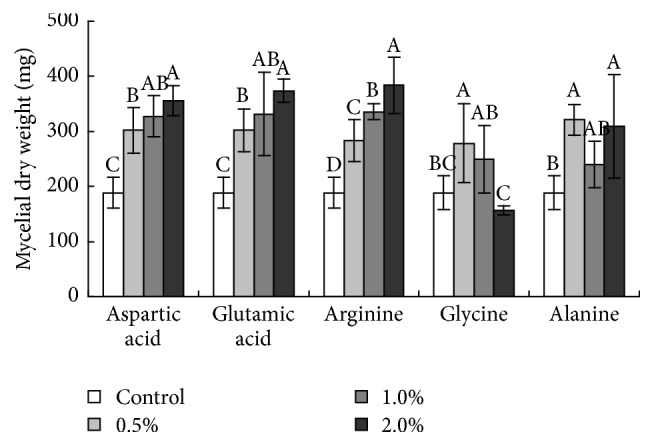
Effects of amino acids on* A. flavus* growth. The data represent the mean ± SD. Letters indicate statistically significant differences (*P* < 0.05) for each amino acid, and all treatments consisted of five replicates.

**Figure 4:**
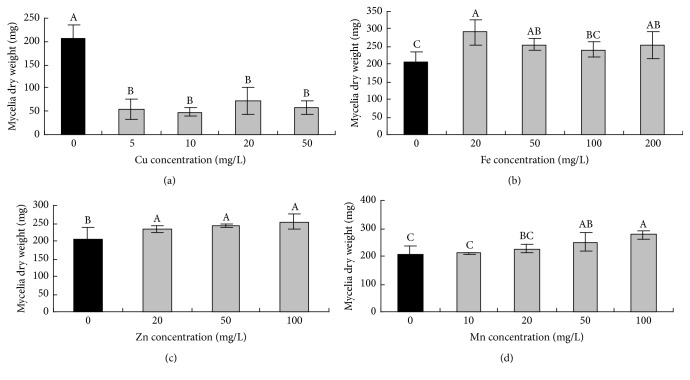
Effects of copper (a), iron (b), zinc (c), and manganese (d) on* A. flavus* growth. Each treatment was grown as described in Section 2. The data represent the mean ± SD. Different superscript letters represent significant differences at *P* < 0.05, and all treatments consisted of five replicates.

The dry weights of the starch samples indicated that when more starch was present, mycelial growth was enhanced (data not shown). The dose relationship between the mycelial growth and soluble sugars revealed that higher concentrations of soluble sugars resulted in improved mycelial growth. The growth-promoting effect of stachyose was obvious, and 3.0 and 6.0% stachyose increased mycelial growth more than 2-fold compared to the other soluble sugars (Figure 2). The effects of amino acids on* A. flavus* were also evaluated (Figure 3). Glutamic acid, aspartic acid, arginine, and alanine stimulated mycelial growth as their concentrations increased. Low concentrations of glycine promoted mycelial growth, but no significant effect was observed with 2.0% glycine. All studied trace elements, except copper, were able to promote* A. flavus* mycelial growth to varying degrees. In contrast, copper significantly reduced mycelial growth at four different concentrations (Figure 4).

## 4. Discussion

There is a close relationship between nutrients and AFB_1_ production in different grains. This study verified that lipids modulate AFB_1_ production by* A. flavus* NRRL 3357. Removal of lipids from ground substrate significantly reduced the substrate's potential for AFB_1_ production. Furthermore, reconstituting the defatted substrates with corn oil restored the AFB_1_ production. In addition, full-fat substrates, which contain abundant oil, can significantly stimulate AFB_1_ production. These results were consistent with those of previous studies, which provided evidence that oil-rich crops are often infected by toxigenic fungi and that lipid contents in seeds contribute to determining the severity of AFB_1_ contamination [10, 11, 28, 35].

Although lipids are one of the most important factors supporting AFB_1_ biosynthesis, other nutrients also play key roles. There were big differences between the nutrients composition and content in six defatted substrates. This study found that different nutrients exert different effects on AFB_1_ production by* A. flavus* NRRL 3357 and that sucrose, glucose, maltose, arginine, glutamic acid, aspartic acid, and zinc significantly stimulated AFB_1_ production by* A. flavus* NRRL 3357. In fact, soluble sugar utilization drives AFB_1_ biosynthesis. In this study, when the soluble sugar concentration was increased, the AFB_1_ concentration also increased. When the concentrations reached 3.0% and 6.0%, soluble sugars substantially promoted AFB_1_ production, especially sucrose, glucose, and maltose. Similarly, Abdollahi and Buchanan [36] found that glucose and sucrose could induce AFB_1_ production. Manda et al. [37] also found that glucose and sucrose were positively correlated with total aflatoxins and also observed a significant positive correlation between AFB_1_ and sucrose. Furthermore, this study found that the contents of six soluble sugars, especially sucrose, were abundant in defatted corn germ, soybean, and peanut and that these contents correlated with AFB_1_ contamination. In addition, Uppala et al. [38] revealed that increasing the sugar content (specifically sucrose) of the media resulted in greater AFB_1_ production by* A. flavus*. Thus, sucrose, glucose, and maltose readily support AFB_1_ biosynthesis in liquid media and may also play significant roles in the AFB_1_ contamination of grain substrates. In six substrates, the content of glutamic acid was the highest, followed by aspartic acid and arginine, and these three amino acids were observed to promote AFB_1_ production by* A. flavus* NRRL 3357. Furthermore, the concentrations of glutamic acid and aspartic and arginine acid in defatted peanut and soybean were higher than those in other defatted substrates. Although Mellon et al. [12] indicated that* A. flavus* does not appear to favour the use of storage proteins as a carbon substrate and that storage proteins are the last to be utilized by fungi [28, 39], our results revealed that glutamic acid and aspartic acid could induce AFB_1_ production; particularly, the study found a new factor, arginine acid, significantly stimulated AFB_1_ production. This discrepancy may be attributed to the different experimental conditions and substrates used. In addition to soluble sugars and amino acids, trace elements also have a substantial effect on AFB_1_ production. Zinc significantly stimulated AFB_1_ biosynthesis. Similarly, Gupta and Venkitasubramanian [40] reported that the addition of zinc to both autoclaved and nonautoclaved soybeans promoted aflatoxin production and indicated that Zn^2+^ is specifically required for aflatoxin biosynthesis by* A. flavus* [41, 42]. Furthermore, at least twenty enzymes have been found to be zinc dependent [43], which may partly account for its key role in AFB_1_ biosynthesis. Thus, analysing the trace elements revealed that defatted corn germ contained abundant zinc, followed by defatted soybean and peanut; however, the contents in maize and corn endosperm were very low, and these values were closely correlated with AFB_1_ contamination. Thus, zinc content may be one of the main causes underlying the varying degrees of contamination of these grains by* A. flavus*.

Compared to the seven nutrients mentioned above, other nutrients did not significantly stimulate AFB_1_ production by* A. flavus* NRRL 3357 (fructose, raffinose, stachyose, alanine, and glycine) and may even inhibit AFB_1_ production (iron, copper, manganese, and starch). Raffinose and fructose do not clearly stimulate AFB_1_ production, which is in contrast to findings obtained by Mellon and Cotty [44] and Manda et al. [37], most likely because of the different strains and medium used. In addition, Tiwari et al. [45] found that iron, copper, and manganese sulfate decreased the aflatoxin production to different levels, consistent with our results. While enhanced aflatoxin production was previously observed by Lijinsky [46] in endosperm and defatted corn supplemented with Mn^2+^ and Cu^2+^, this result was not consistent with those obtained in our study, possibly because of the use of in vitro experiments and trace element ions in our study. In addition, most trace elements present in grains occur in bound forms. Although starch is abundant in corn, corn endosperm, and wheat, it does not to appear induce AFB_1_ biosynthesis. It is possible that the high starch content will change the characteristics of the ground substrates and that the hygroscopic nature of starch granules impedes water acquisition by the fungus [12].

These nutrients not only affected AFB_1_ production by* A. flavus* NRRL 3357 but also modulated* A. flavus* growth. Starch and all types of soluble sugars, amino acids, and trace elements increased* A. flavus* growth, except copper, and stachyose exhibited the most significant effect. Uppala et al. [38] reported that increasing the sugar content in the media increased the mycelial growth. Moreover, many amino acids, such as asparagine, proline, and hydroxyproline, supported better mycelial growth [18]. Additionally, because increased mycelial growth is generally associated with increased toxin production [18], mycelial growth may increase the AFB_1_ production capacity. Thus, these results are consistent with reports of AFB_1_ contamination in various substrates. Indeed, soluble sugars, trace elements, and amino acids are abundant in defatted corn germ, peanut, and soybean.* A. flavus* is a causal agent of aspergillosis and the second-most-common pathogen responsible for invasive and noninvasive aspergillosis [47]. In addition, farmers infected by breathing in spores from contaminated foods and feeds have been reported worldwide [48] and animals can also be infected by* A. flavus* [49]. Thus, mycelial growth not only promotes AFB_1_ production but also threatens human and animal health.

In conclusion, the study systematically researched the effects of nutrients on AFB_1_ accumulation and found the relationship between the various nutrients in grains and AFB_1_ contamination levels in various grains and isolate tissues. In addition to lipids, other nutrients in substrate also play key roles in AFB_1_ biosynthesis and mycelial growth. Maltose, glucose, sucrose, arginine, glutamic acid, aspartic acid, and zinc significantly stimulate AFB_1_ biosynthesis. All types of soluble sugars, amino acids, and trace elements increase mycelial growth, except copper, as evidenced by the strong relationship observed for stachyose. According to the nutrient contents and their enhancing abilities for AFB_1_ production by* A. flavus* NRRL 3357 and* A. flavus* growth, in addition to lipids, sucrose, stachyose, glutamic acid, and zinc may play key roles in the differential AFB_1_ contamination of various grains and isolate tissues. Particularly, we found that a new nutrient (arginine) of grains significantly stimulates AFB_1_ production and a new nutrient (stachyose) significantly stimulates* A. flavus* growth. Although saccharides are utilized by* A. flavus* first, followed by triglycerides and finally protein [12, 28, 39], the interactions between these nutrients remain poorly understood. Thus, further studies should focus on the effects of the interactions between these nutrients on AFB_1_ production by* A. flavus*.

## Figures and Tables

**Table 1:** Effects of corn oil and different substrates on AFB_1_ production by *A. flavus *NRRL3357.

Aflatoxin B_1_^D^	Defatted substrates	Defatted substrates + corn oil^E^	Full-fat substrates
Soybean	8.15 ± 2.13^B^	52.06 ± 8.31^A^	66.26 ± 11.95^A^
Peanut	3.04 ± 0.33^C^	22.55 ± 7.07^A^	10.14 ± 1.07^B^
Corn	0.04 ± 0.02^B^	9.46 ± 0.56^A^	0.12 ± 0.01^B^
Wheat	0.03 ± 0.00^B^	14.87 ± 5.11^A^	0.11 ± 0.05^B^
Corn endosperm	0.41 ± 0.11^B^	5.49 ± 1.54^A^	0.17 ± 0.04^B^
Corn germ	6.82 ± 1.14^C^	33.03 ± 3.49^B^	48.21 ± 7.29^A^

^A–C^Means within rows followed by the same letter are not significantly different at *P* ≥ 0.05.

^D^Mean aflatoxin B_1_ is expressed in milligrams per kilogram (*n* = 5).

^E^An average of 10% corn oil was added to defatted substrates.

**Table 2:** Starch, trace element, soluble sugar, and amino acid contents of different substrates.

Nutrients	Defatted soybean	Defatted peanut	Defatted wheat	Defatted corn	Defatted corn endosperm	Defatted corn germ
Starch content (%)						
Starch	5.26 ± 0.94^E^	7.01 ± 0.71^E^	67.22 ± 2.87^C^	72.21 ± 1.97^B^	82.19 ± 1.11^A^	28.12 ± 0.76^D^
Trace elements contents (mg/kg)						
Copper	25.33 ± 9.41^AB^	34.63 ± 2.44^A^	12.12 ± 1.90^CD^	6.89 ± 0.35^D^	6.79 ± 1.70^D^	19.86 ± 3.21^BC^
Iron	93.47 ± 0.22^B^	64.18 ± 7.68^C^	83.97 ± 8.51^B^	21.95 ± 4.15^D^	6.23 ± 3.36^D^	145.05 ± 11.31^A^
Zinc	51.90 ± 4.07^C^	84.40 ± 10.88^B^	39.14 ± 3.23^D^	18.96 ± 1.52^E^	12.47 ± 0.91^E^	107.47 ± 1.94^A^
Manganese	36.19 ± 0.65^B^	59.86 ± 1.92^A^	60.30 ± 0.15^A^	5.28 ± 0.18^D^	2.13 ± 0.22^E^	30.54 ± 0.27^C^
Soluble sugar concentrations (mg/g)						
Fructose	3.89 ± 1.65^BC^	11.12 ± 2.06^A^	1.94 ± 0.06^C^	7.72 ± 0.18^AB^	1.88 ± 0.24^C^	9.67 ± 2.65^A^
Glucose	1.61 ± 0.52^B^	4.92 ± 1.12^B^	4.01 ± 0.13^B^	4.95 ± 0.12^B^	7.21 ± 0.07^B^	13.75 ± 4.27^A^
Sucrose	31.17 ± 5.05^A^	32.5 ± 0.46^A^	2.24 ± 0.12^B^	2.15 ± 0.33^B^	5.07 ± 0.1^B^	42.36 ± 9.19^A^
Maltose	0.63 ± 0.14^C^	0.54 ± 0.07^C^	4.10 ± 0.35^B^	4.05 ± 0.35^B^	7.96 ± 0.60^A^	1.66 ± 0.28^C^
Raffinose	3.32 ± 0.41^B^	2.08 ± 0.27^B^	3.00 ± 0.05^B^	1.11 ± 0.17^B^	1.16 ± 0.03^B^	10.71 ± 2.62^A^
Stachyose	34.71 ± 7.39^A^	6.78 ± 0.98^B^	0.36 ± 0.06^B^	ND^B^	ND^B^	0.72 ± 0.10^B^
Amino acid contents (%)						
Aspartic acid	6.3 ± 0.38	7.70 ± 0.23	0.74 ± 0.04	0.54 ± 0.01	0.51 ± 0.15	1.81 ± 0.05
Glutamic acid	10.56 ± 0.57	13.89 ± 0.39	4.56 ± 0.24	1.72 ± 0.01	1.93 ± 0.20	3.33 ± 0.05
Glycine	2.46 ± 0.10	3.35 ± 0.15	0.55 ± 0.03	0.34 ± 0.00	0.30 ± 0.02	1.16 ± 0.02
Arginine	3.81 ± 0.18	6.95 ± 0.18	0.65 ± 0.04	0.43 ± 0.00	0.37 ± 0.03	1.73 ± 0.04
Alanine	2.58 ± 0.11	2.97 ± 0.06	0.51 ± 0.03	0.61 ± 0.01	0.66 ± 0.01	1.37 ± 0.01
Protein level	45.11 ± 2.53	55.45 ± 1.82	12.25 ± 0.37	8.36 ± 0.09	8.60 ± 1.20	19.47 ± 0.42

^A–E^Means within rows followed by the same letter are not significantly different at *P* ≥ 0.01 according to Duncan's multiple range test (*n* = 3).

**Table 3:** Effect of soluble sugars on AFB_1_ production.

Aflatoxin B_1_^D^	Concentration of soluble sugars (%)			
	0.5	1.0	3.0	6.0
Fructose	46.92 ± 11.30^B^	7.54 ± 0.83^B^	5.41 ± 0.56^B^	1137.56 ± 308.79^A^
Glucose	28.77 ± 3.33^B^	8.92 ± 0.77^B^	5664.33 ± 570.70^B^	35860.57 ± 7028.16^A^
Sucrose	4.63 ± 1.26^B^	4.28 ± 0.60^B^	39782.61 ± 5896.87^A^	4471.97 ± 442.68^B^
Maltose	2.84 ± 0.85^C^	2.34 ± 0.33^C^	23687.29 ± 5777.01^B^	74848.68 ± 8422.06^A^
Raffinose	5.70 ± 1.13^B^	2.76 ± 0.65^B^	208.27 ± 24.28^A^	5.50 ± 1.21^B^
Stachyose	17.02 ± 1.22^B^	10.08 ± 1.39^B^	5426.44 ± 292.49^A^	120.61 ± 16.55^B^

^A–C^Mean values with different letters for each soluble sugar are significantly different (*P* < 0.05).

^D^Aflatoxin B_1_ values are the average of five replications with standard deviation in ng/30 mL.

**Table 4:** Effect of trace elements on AFB_1_ production.

Aflatoxin B_1_^D^	Control	Concentration of trace elements (mg/L)					
		5	10	20	50	100	200
Copper	238.35 ± 36.61^A^	4.39 ± 0.29^B^	4.90 ± 0.59^B^	3.00 ± 0.94^B^	4.31 ± 0.08^B^	—	—
Iron	238.35 ± 36.61^A^	—	—	2.39 ± 0.08^B^	2.71 ± 1.21^B^	0.36 ± 0.23^B^	0.73 ± 0.20^B^
Zinc	238.35 ± 36.61^C^	—	—	954.95 ± 21.15^BC^	1232.08 ± 312.71^B^	4733.88 ± 795.24^A^	—
Manganese	238.35 ± 36.61^A^	—	1.75 ± 0.14^B^	17.87 ± 3.53^B^	29.30 ± 4.87^B^	10.00 ± 3.18^B^	—

—: concentration not studied.

^A–C^Mean values with different letters for each trace element are significantly different (*P* < 0.05).

^D^Aflatoxin B_1_ values are the average of five replications with standard deviation in ng/30 mL.

## References

[1] Dhingra O. D., Jham G. N., Rodrigues F. Á., Silva G. J., Costa M. L. N. (2009). Fumigation with essential oil of mustard retards fungal growth and accumulation of ergosterol and free fatty acid in stored shelled groundnuts. *Journal of Stored Products Research*.

[2] Astoreca A. L., Dalcero A. M., Pinto V. F., Vaamonde G. (2011). A survey on distribution and toxigenicity of *Aspergillus* section *Flavi* in poultry feeds. *International Journal of Food Microbiology*.

[3] Perrone G., Haidukowski M., Stea G. (2014). Population structure and Aflatoxin production by *Aspergillus Sect. Flavi* from maize in Nigeria and Ghana. *Food Microbiology*.

[4] Ma X., Wang W., Chen X. (2014). Selection, identification, and application of Aflatoxin B1 aptamer. *European Food Research and Technology*.

[5] Li X. Y., Zhao L. H., Fan Y. (2014). Occurrence of mycotoxins in feed ingredients and complete feeds obtained from the Beijing region of China. *Journal of Animal Science and Biotechnology*.

[6] International Agency for Research on Cancer (IARC) (2002). *Some Naturally Occurring Substances: Food Items and Constituents, Heterocyclic Aromatic Amines and Mycotoxins*.

[7] Kumar A., Shukla R., Singh P., Dubey N. K. (2010). Chemical composition, antifungal and antiaflatoxigenic activities of *Ocimum sanctum* L. essential oil and its safety assessment as plant based antimicrobial. *Food and Chemical Toxicology*.

[8] Fanelli C., Fabbri A. A. (1989). Relationship between lipids and aflatoxin biosynthesis. *Mycopathologia*.

[9] Keller N. P., Butchko R. A. E., Sarr B., Phillips T. D. (1994). A visual pattern of mycotoxin production in maize kernels by *Aspergillus* spp.. *Phytopathology*.

[10] Wilson R. A., Calvo A. M., Chang P.-K., Keller N. P. (2004). Characterization of the *Aspergillus parasiticus* Δ12-desaturase gene: a role for lipid metabolism in the Aspergillus-seed interaction. *Microbiology*.

[11] Chang P.-K., Wilson R. A., Keller N. P., Cleveland T. E. (2004). Deletion of the Δ12-oleic acid desaturase gene of a nonaflatoxigenic *Aspergillus parasiticus* field isolate affects conidiation and sclerotial development. *Journal of Applied Microbiology*.

[12] Mellon J. E., Dowd M. K., Cotty P. J. (2005). Substrate utilization by *Aspergillus flavus* in inoculated whole corn kernels and isolated tissues. *Journal of Agricultural and Food Chemistry*.

[13] Ma H., Zhang N., Sun L., Qi D. (2014). Effects of different substrates and oils on aflatoxin B_1_ production by *Aspergillus parasiticus*. *European Food Research and Technology*.

[14] Woloshuk C. P., Cavaletto J. R., Cleveland T. E. (1997). Inducers of aflatoxin biosynthesis from colonized maize kernels are generated by an amylase activity from *Aspergillus flavus*. *Phytopathology*.

[15] Fakhoury A. M., Woloshuk C. P. (1999). Amy1, the *α*-amylase gene of *Aspergillus flavus*: involvement in aflatoxin biosynthesis in maize kernels. *Phytopathology*.

[16] Fakhoury A. M., Woloshuk C. P. (2001). Inhibition of growth of *Aspergillus flavus* and fungal *α*-amylases by a lectin-like protein from *Lablab purpureus*. *Molecular Plant-Microbe Interactions*.

[17] Davis N. D., Diener U. L. (1968). Growth and aflatoxin production by *Aspergillus parasiticus* from various carbon sources. *Applied Microbiology*.

[18] Payne G. A., Hagler W. M. (1983). Effect of specific amino acids on growth and aflatoxin production by *Aspergillus parasiticus* and *Aspergillus flavus* in defined media. *Applied and Environmental Microbiology*.

[19] Reddy T. V., Viswanathan L., Venkitasubramanian T. A. (1971). High aflatoxin production on a chemically defined medium. *Applied Microbiology*.

[20] Lillehoj E. B., Garcia W. J., Lambrow M. (1974). *Aspergillus flavus* infection and aflatoxin production in corn: influence of trace elements. *Journal of Applied Microbiology*.

[21] Stossel P. (1986). Aflatoxin contamination in soybeans: role of proteinase inhibitors, zinc availability, and seed coat integrity. *Applied and Environmental Microbiology*.

[22] Cuero R., Ouellet T., Yu J., Mogongwa N. (2003). Metal ion enhancement of fungal growth, gene expression and aflatoxin synthesis in *Aspergillus flavus*: RT-PCR characterization. *Journal of Applied Microbiology*.

[23] Schmidt-Heydt M., Abdel-Hadi A., Magan N., Geisen R. (2009). Complex regulation of the aflatoxin biosynthesis gene cluster of *Aspergillus flavus* in relation to various combinations of water activity and temperature. *International Journal of Food Microbiology*.

[24] Herrero-Garcia E., Garzia A., Cordobés S., Espeso E. A., Ugalde U. (2011). 8-Carbon oxylipins inhibit germination and growth, and stimulate aerial conidiation in *Aspergillus nidulans*. *Fungal Biology*.

[25] Kartika I. A., Yuliani S., Kailaku S. I., Rigal L. (2012). Moisture sorption behaviour of jatropha seed (*Jatropha curcas*) as a source of vegetable oil for biodiesel production. *Biomass and Bioenergy*.

[26] ISO (1999). *ISO 6496: Animal Feeding Stuffs—Determination of Moisture and Other Volatile Mater Content*.

[27] GB/T (2008). Determination of starch in foods. *GB/T*.

[28] Mellon J. E., Cotty P. J., Dowd M. K. (2000). Influence of lipids with and without other cottonseed reserve materials on aflatoxin B1 production by *Aspergillus flavus*. *Journal of Agricultural and Food Chemistry*.

[29] GB/T 18246-2000 (2000). *Determination of Amino Acids in Feeds*.

[30] Gratzfeld-Huesgen A. Sensitive and reliable amino acid analysis in protein hydrolysates using the Agilent 1100 series HPLC. Agilent Technology Literature Library. http://www.chem.agilent.com/Library/technicaloverviews/Public/59685658.pdf.

[31] ISO (2000). Animal feeding stuffs-determination of the contents of calcium, copper, iron, magnesium, manganese, potassium, sodium and zinc-method using atomic absorption spectrometry. *ISO*.

[32] Wilkinson J. R., Yu J., Bland J. M., Nierman W. C., Bhatnagar D., Cleveland T. E. (2007). Amino acid supplementation reveals differential regulation of aflatoxin biosynthesis in *Aspergillus flavus* NRRL 3357 and *Aspergillus parasiticus* SRRC 143. *Applied Microbiology and Biotechnology*.

[33] Trucksess M. W., Stack M. E., Nesheim S., Albert R. H., Romer T. R. (1994). Multifunctional column coupled with liquid chromatography for determination of aflatoxins B1, B2, G1, and G2 in corn, almonds, Brazil nuts, peanuts, and pistachio nuts: collaborative study. *Journal of AOAC International*.

[34] Abdel-Hadi A. M., Caley D. P., Carter D. R. F., Magan N. (2011). Control of aflatoxin production of *Aspergillus flavus* and *Aspergillus parasiticus* using RNA silencing technology by targeting aflD (nor-1) gene. *Toxins*.

[35] Dall'Asta C., Falavigna C., Galaverna G., Battilani P. (2012). Role of maize hybrids and their chemical composition in Fusarium infection and fumonisin production. *Journal of Agricultural and Food Chemistry*.

[36] Abdollahi A., Buchanan R. L. (1981). Regulation of aflatoxin biosynthesis: induction of aflatoxin production by various carbohydrates. *Journal of Food Science*.

[37] Manda A., Bodapati P. N., Rachaputi N. C., Wright G., Fukai S. Aflatoxins and their relationship with sugars in peanut (*Arachishypogaea* L.).

[38] Uppala S. S., Bowen K. L., Woods F. M. (2013). Pre-harvest aflatoxin contamination and soluble sugars of peanut. *Peanut Science*.

[39] Mellon J. E., Dowd M. K., Cotty P. J. (2002). Time course study of substrate utilization by *Aspergillus flavus* in medium simulating corn (*Zea mays*) kernels. *Journal of Agricultural and Food Chemistry*.

[40] Gupta S. K., Venkitasubramanian T. A. (1975). Production of aflatoxin on soybeans. *Journal of Applied Microbiology*.

[41] Mateles R. I., Adye J. C. (1965). Production of aflatoxins in submerged culture. *Applied Microbiology*.

[42] Marsh P. B., Simpson M. E., Trucksess M. W. (1975). Effects of trace metals on the production of aflatoxins by *Aspergillus parasiticus*. *Journal of Applied Microbiology*.

[43] Parisi A. F., Vallee B. L. (1969). Zinc metalloenzymes: characteristics and significance in biology and medicine. *The American Journal of Clinical Nutrition*.

[44] Mellon J. E., Cotty P. J. (1999). Raffinose content may influence cottonseed susceptibility to aflatoxin contamination. *Journal of the American Oil Chemists' Society*.

[45] Tiwari R. P., Mittal V., Bhalla T. C., Saini S. S., Singh G., Vadehra D. V. (1986). Effect of metal ions on aflatoxin production by *Aspergillus parasiticus*. *Folia Microbiologica*.

[46] Lijinsky W. (1970). The preparation of aflatoxins labeled with tritium. *Journal of Labelled Compounds*.

[47] Krishnan S., Manavathu E. K., Chandrasekar P. H. (2009). *Aspergillus flavus*: an emerging non-*fumigatus Aspergillus* species of significance. *Mycoses*.

[48] Adhikari A., Sen M. M., Gupta-Bhattacharya S., Chanda S. (2004). Volumetric assessment of airborne fungi in two sections of a rural indoor dairy cattle shed. *Environment International*.

[49] Hedayati M. T., Pasqualotto A. C., Warn P. A., Bowyer P., Denning D. W. (2007). *Aspergillus flavus*: human pathogen, allergen and mycotoxin producer. *Microbiology*.

